# Mississippi Valley Dental Association

**Published:** 1885-05

**Authors:** E. G. Betty


### MISSISSIPPI VALLEY DENTAL ASSOCIATION.

FORTY-FIRST ANNUAL MEETING AT CINCINNATI, MARCH 4, 5, AND 6, 1885.

REPORTED FOR THE INDEPENDENT PRACTITIONER BY E. G. BETTY, D. D. S.

(Concluded from page 203.)

SECOND DAY—MORNING SESSION.

The first subject was passed, and the second, "Recent Theories Concerning Caries of the Teeth" announced. All the members not having arrived, and it not being advisable to pass the subject without a hearing, Dr. Betty moved that in the meanwhile Dr. W. D. Kempton be allowed to read a paper on "How to Improve Tooth Structure," of which the following is a synopsis:

Cervantes said: "I had rather they had torn off an arm, provided it were not the sword arm, for thou must know, Sancho, that a mouth without teeth is like a mill without a stone, and that a diamond is not as precious as a tooth." There has been and is now said a great deal upon this subject, and what I may say will not be new to you. In the midst of discussions as to what method and material are best for filling teeth, we hear it said that the teeth are poor in quality, owing to a lack of phosphates in the food, and that these can be supplied by an oatmeal and graham flour diet. But this seldom seems to meet the approval of the patient, and the stomach soon tires of it. The fault lies in the bad cooking of the cereals, as the oatmeal, cracked wheat, etc., are put in a great quantity of water, and boiled and stirred until the resulting mass is anything but palatable. It is better to cook them in a water bath, with but little water, and thus no flavor is lost and the food can be taken with relish. Defective assimilation is often responsible for poor teeth, even though the diet be good. Assimilation is that process which turns grass into hair, when eaten by cattle, and into wool by the sheep; it is to the body what the workmen are to the building. Sunlight stimulates assimilation, and we should have more of it in our houses, upon the same principle that prompts the florist to supply abundance of it for his hothouse. Bodily exercise creates a demand in the tissues for their proper nourishment, and that exercise is best that is combined with pleasure. Sunlight, fresh air, and exercise are the factors that make good tissues out of good food, and they are all as necessary for the production of good tooth structure as they are for the body generally. A local condition is the free use of the teeth, as an organ well used develops better than one not often called upon, so that the mania for chewing gum among children is rather to be encouraged than condemned. Amongst unfavorable influences for the proper development and nurture of the teeth is heredity, but that we cannot control. Over-stimulation of the young brain in mental studies and tasks at school is to be frowned down, as stimulation of brain in the young is always at the expense of their bones and muscles, the teeth being like chalk and the muscles almost *nil.* In conclusion, the essayist urged that the best way to induce the formation of good teeth is to nourish the body generally, and the organs of mastication will share in the summing up of the result.

Dr. C. M. Wright then read a very short paper upon the "Development Hypothesis," in which he said that if this theory be true, and if it can be shown that any existing species, animal or vegetable, when placed under conditions different from its preceding ones immediately begins to undergo certain changes of structure, fitting it for the new conditions, then it seems that dental caries can be placed in its proper relation in the economy of nature. As man and his nervous system developed, and as the facial angle became more perpendicular, the teeth lost their use as weapons of defense, and as a means of procuring food, and the gradual change caused them to deteriorate. This may account for the existence of the dentist. This being the case, how can we save teeth by any of our means and methods when they are doomed to go? The law of the survival of the fittest applies to teeth, as well as any other organ or tissue.

*Dr. A. W. Harlan*—I listened to Dr. Kempton's paper with interest, and I think we cannot overestimate the value of outdoor exercise. The rules for food, light and exercise must be followed, for if they are not, the teeth suffer in common with other organs of the body. Correct principles of hygiene have more to do with formation of good dentine than does the administration of specific foods. I do not take the same gloomy view of the decay of the teeth in the present generation that many do, as our teeth compare favorably with those of prehistoric times, and even those of two or three hundred years ago. We see many more than our forefathers did, and try to save the whole thirty-two, while they were content with eighteen or twenty. The large quantities of filling materials we use attest our efforts in the conservation of the teeth, and it is not uncommon to see teeth doing good service that were filled thirty or forty years ago. Our high aim in the future is to discover causes, that we may more intelligently employ preventive means.

*Dr. Wright*—I am sorry if my little paper conveyed the impression that it is useless to attempt to save teeth, as we do save millions. I only took the broad view that in the future they are doomed.

*Dr. Taft*—I fail to see the use of discussing what will happen five million years hence, and the retrograding process Dr. Wright has mentioned. Our work is with the teeth of to-day; how to save them when diseased, and what to do to assist in the best formation of them. Specialists confined to one subject are apt to overrate its importance. You cannot specify any course that will develop one organ without building up the whole body.

*Dr. Wright*—I am sorry that Prof. Taft does not care for harmony and the "law of nature." I thought we might get down to something positive if we took a broad and liberal view of the whole field of development.

*Prof. Taft*—I deny that the matter to which Dr. Wright referred is a law of nature.

*Dr. Berry*—I regret exceedingly that Dr. Miller is not with us, that he might give us a thorough exposition of his theory of dental caries.

*Dr. H. A. Smith*—Dr. Berry has called for something on the theories of caries. There is a more recent theory than that of Miller. It is that of Black. His theory is, that what we have called abrasion near the margin of the gum is caused by the secretion of a solvent, which causes the wasting away of tissue, notably upon inferior cuspids. Is it a ferment, or what is it? The wasting occurs one-third the way up the tooth, and against gravity. Another peculiarity is, that the nearer the pulp is approached the softer is the tissue. I would like to have this explained.

*Dr. McKellops*—This question came up some time ago, at a social gathering at which Dr. Black was present. I see such cases all the time. One lady, aged thirty, had softening and wasting of the molars, although she had good work done. She came to me after she had been married and become a mother. All the molars and bicuspids had lost their enamel without decay. Another instance was that of a gentleman and his wife. Nothing seems to do so much good in these cases as oxy-phosphate of zinc.

*Dr. H. A. Smith*—Does the withdrawal of the nutrition of the dentine cause it to soften and waste away ? Is it right to fill and promise immunity ?

The subject was passed on motion, and the third subject, "New Remedies for the Treatment of Dental and Oral Diseases," announced. Dr. Harlan, of Chicago, read a paper which in substance is as follows:

Cocaine Hydrochlorate.—Judging from an experience of several months with this remedy, in two and four per cent. aqueous solutions, it is nearly valueless for obtunding sensitive dentine in superficial cavities. A ten per cent.. aqueous solution is effective when kept in the cavity for ten minutes. A ten per cent. solution in eugenol is still more effective, and I prefer it for deep-seated cavities. For extirpation of the pulp, a ten per cent. solution in ether is preferable, as the rapid evaporation of the ether also assists in the production of anæsthesia. An injection of a four per cent. aqueous solution in the pyorrhœal pockets, prevents pain from the removal of small deposits from the roots. Cocaine is a styptic, due to a peculiar tannate that resides in the leaf of the plant. It is possible to extract three or four teeth painlessly if a solution is deeply injected near the mental foramen, as the dental nerve and its branches appear to lose sensation.

Eugenol is obtained from the oil of cloves. It is eugenic acid, and may be called the active principle. It will supersede all other remedies for the treatment of blind abscesses ; I use it in preference for that purpose. No other drug is so agreeable to the patient. It is a potent germicide. It is prepared for use by dissolving in some such oil as cinnamon, sassafras, or wintergreen. Iodoform made into a creamy paste with eugenol makes an elegant and efficient dressing for an exposed or nearly exposed pulp. The same paste may be used in pockets of pyorrhœa, and will reduce the inflammation and pain. It will also relieve severe odontalgia when prepared thus:

℞.—Eugenol and oil of cinnamon, ʒ ij. each;

Cryst. carbolic acid, ʒ i.

Mix and apply on cotton.

SANITAS is the oxidized oil of turpentine, and contains ten to twelve volumes of $H_2$ $O_2$, which is permanently stored up for use in the future. I use it for treatment of all blind abscesses, and inject it into pyorrhœal pockets when an astringent is not indicated. In the latter case it is mixed with some of the essential oils, sassafras, etc., in proportion of one to three of Sanitas. It is not poisonous, except in large doses. Peroxide of hydrogen and thymol, both contained in Sanitas, are powerful germicides, and are not poisonous. When opening into a pulp chamber I always do it under Sanitas oil.

CANNABIS INDICA. Of this I make a tincture of ten ounces of the tops to one pint of alcohol. This is much stronger than the officinal tincture. I have removed one pulp painlessly after the application of it, and have also obtunded the sensibility of dentine with it.

Dr. J. S. Cassidy, of Covington, Ky., read a paper on "Cocaine Chloride."

SYNOPSIS.—It is worthy of trial, and I will give a few cases in which a four cent. solution was used: First—Unmarried female, æt. twenty-five; lymphatic temperament. Applied the solution. No pain in the dentine at the end of ten minutes. Second case—Gentleman; tobacco chewer. No effect produced. Third—Child; complained of pain in adjusting the clamp. Cocaine relieved all pain. Fourth—Female, æt. twenty; superficial cavity, very sensitive. Applied cocaine; no pain. Fifth case—Pulp partly dead. Bathed with cocaine without effect. Sixth—Skeptical scientist. Applied cocaine to a sensitive tooth. No pain on excavation.

Does the effect of cocaine depend on the idiosyncrasy of the patient, or must there exist some local circumstances that resist its anæsthetic effect? Dr. E. G. Betty, of Cincinnati, in an article in the February number of the *Register*, says that vascularity seems to be an essential condition, and that its effectiveness is due to its ability to produce anæmia. On the other hand, Dr. Arnold, of Ironton, O., presents in the same number of the *Register* several cases in which he produced perfect anæsthesia of the dentine by the use of this agent. It seems that it acts on non-vascular tissue by producing a paralysis of peripheral sensory material. As it produces no cauterization it cannot always be successfully used on sensitive dentine. It is possible that some other salt than the chloride will be used by the dentist. I am of the opinion that we ought not to call the salt hydrochlor*ate*, as a salt whose name ends in *ide* contains an element for its acidulous base. Consequently a salt formed by hydrochloric acid should end in *ide*. Others have called the salt "chloride" before, as I see in some recent publications, and it is not necessary to prefix *hydro*.

*Dr. Will. Taft.*—I procured some cocaine when it was first introduced to our notice, and have been using it ever since, though for obtunding sensitive dentine it is of no account. Upon soft tissues it produces anæsthesia, and is quite valuable for preventing nausea in taking impressions.

*Dr Wright*—I would like to ask Dr. Harlan if cannabis indica should be classed with cocaine? It has been in use in England of late.

*Dr. Harlan*—I do not think it should, as it does not prevent tissue waste, as does cocaine. Some gentleman, of a commercial tendency, has tried to patent a method of extracting teeth by the use of cannabis indica. I have no use for such a person. He uses a weak aqueous solution, hot, dips the forceps in it, and extracts quickly. That is all there is in it. If we could obtain the active principle, cannabin, I think we would no doubt get the same result as with cocaine. I have experimented with theine, caffeine and theo-bromine, but obtained no results. The alkaloid cocaine is soluble in 700 parts of water, but the salts are very soluble. In one case I made a rope of cotton, saturated it with a four per cent. solution, tied it around the neck of a tooth, and after ten minutes the tooth was extracted without pain. In cases requiring the removal of necrosed bone from the gingival margin, painting the gum with a four per cent. solution relieves all pain.

*Dr. McKellops*—I have tried the cocaine in this form: Cocaine, one and one-half grains, dissolved in one-half drachm each of alcohol and chloroform. This gives a much better result than the aqueous solution. I have also used the cannabis indica with success in the extraction of teeth.

AFTERNOON SESSION.

Dr. A. O. Rawls read an exhaustive paper on "Pyorrhœa Alveolaris," giving its history and such of the etiology as is known, symptoms, varieties and treatment, both surgical and therapeutical.

*Dr. Sage*—I have been in the habit of using Listerine for the treatment of this affection, and like it very much.

*Dr. A. Berry*—In treating this disease look well to the constitutional condition. The too free use of common salt is undoubtedly a prominent cause of the disease.

*Prof. Taft*—In employing the agents Dr. Harlan described, there are certain principles to guide their use. Is an antiseptic demanded? Then choose the one that seems best adapted to the case. Does the condition require a mere disinfectant? Then out of the list of disinfectants select and apply that which best meets all the symptoms. In considering the application of any agent we should know if it is antiseptic, stimulant, or astringent, or all combined. There is a great difference of opinion as to whether pyorrhœa alveolaris is local or systemic. If the constitution is good, tissues perfect, and nutrition and elimination well carried on, the disease, if present, can then only be local. In a loose, flabby system, with functions indifferently performed, there is a great susceptibility to all kinds of affections. The nomination of the disease means pus oozing from the gums. This may be produced by local irritants, which, if removed, allow the parts to restore themselves. An acrid secretion from the mouth which gets into the pockets, is sufficient to keep up the irritation. The saliva is sometimes so acrid that it burns the skin. Salt produces a change in the tissues and bodies with which it comes in contact. In the system it prevents the free elimination of waste, by preventing its free solution. I would be glad if Dr. Harlan would tell us *how* and *why* some of the agents of which he spoke this morning act, particularly sanitas and eugenol.

*Dr. Harlan*—Sanitas stores up $H_2 O_2$ which, while not generally recognized by dentists, is a germicide.

*Prof. Taft*—For all germs?

*Dr. Harlan*—For all bacterial forms. Sanitas is a true disinfectant. It does not substitute one smell for another. A disinfectant is capable of combining with the gases and other products of decomposition, and so removing it. Sanitas is the oxidized oil of turpentine, and is itself a powerful oxidizer. It does not give up its $H_2 O_2$ until there is a call for it. It combines $H_2 O_2$ with a dressing. A great many who complain of want of success with sanitas can only blame their own carelessness. No outside matter, such as saliva, mucus, etc., should be permitted to enter the pulp chamber. Open it only when dry. If the taste of sanitas is objectionable, then use eugenol. The reputation of the latter as a parasiticide rests upon solid facts. Eugenol is a decided stimulant; it does not come under the head of disinfectants; it does not enter into combination with foul matter and break it up; its composition precludes that ($C_{10} H_{12} O_2$, if memory is correct).

Sanitas remains where it is injected; it cannot be mixed with saliva, though water may dilute it slightly. Iodoform may be disguised with eugenol or oil of peppermint. I prefer to open a pulp chamber under sanitas, then fill the cavity with gutta percha, punctured to permit gas to escape.

THIRD DAY—LAST SESSION.

The third subject was passed and the fourth announced: "Improvements in Prosthetic Dentistry." No papers.

*Dr. McKellops*—I have not made any artificial work for fifteen years. I saw some of Dr. Patrick's bridge work lately, and it is beautiful. It fitted so perfectly that when first put in it turned the gum white with the pressure. This passed away in a short time.

*Dr. H. A. Smith*—It seems to me that the accumulation of debris between the piece and the gum, would result in irritation of the mucous surfaces.

*Dr McKellops*—There is no occasion or opportunity for matter to get under the plate, as it is so perfectly fitted to the parts.

*Dr. H. A. Smith*—That is a remarkable statement, but I will not contradict it.

*Dr. Osmond*—All pulpless teeth to which crowns or pieces are fitted, will in time be pushed from their sockets by deposits, and the plate lifted from the gums.

*Dr. McKellops*—I have also seen some very beautiful work done by Dr. Morrison. One case was half of a molar cemented on, so that there could be no friction. It has been worn for years.

*Dr. Sage*—The advocates of bridge work do not use roots that have deposits on them, but take good teeth. There are so few opportunities for the putting in of bridge work, that I do not think it will come into general use.

*Dr. McKellops*—I would like to report another case from Dr. Morrison. In an irregularity case, an upper front tooth struck against a lower cuspid and caused pain and trouble. He extracted both, shaped the cuspid so as to resemble the upper incisor, building out the sides with gold. He burred out the upper alveolus to form a socket, and placed the remodeled tooth in it. It has now been in about five months, and is as solid as any other tooth in the head.

After the transaction of some miscellaneous business, the following named gentlemen were elected as the officers for the ensuing year:

*President*—A. F. Emminger.

*First Vice-President*—E. G. Betty.

*Second Vice-President*—Otto Arnold.

*Recording Secretary*—A. G. Rose.

*Corresponding Secretary*—W. D. Kempton.

*Treasurer*—F. A. Hunter.

The Association then adjourned, to meet again the first Wednesday in March, 1886.

